# Emerging knock-down resistance in *Anopheles arabiensis* populations of Dakar, Senegal: first evidence of a high prevalence of *kdr*-*e* mutation in West African urban area

**DOI:** 10.1186/s12936-015-0898-6

**Published:** 2015-09-22

**Authors:** Mamadou Ousmane Ndiath, Aurélie Cailleau, Eve Orlandi-Pradines, Paul Bessell, Fréderic Pagès, Jean-François Trape, Christophe Rogier

**Affiliations:** Groupe G4 Institut Pasteur International Network, Institut Pasteur de Bangui, BP 237, Bangui, Central African Republic; Laboratoire de Paludologie et de Zoologie, Campus UCAD-IRD, BP 1386, CP 18524 Dakar, Senegal; Afrique One, Centre Suisse de Recherches Scientifiques, 01 BP 1303, Abidjan, Côte d’Ivoire; UMR 6236, Unité d’entomologie médicale, IRBA antenne Marseille, GSBDD Marseille Aubagne, 111 avenue de la Corse, BP 40026, 13568 Marseille Cedex 2, France; The Roslin Institute, The University of Edinburgh, Easter Bush, Edinburgh, EH25 9RG UK; Institut Pasteur de Madagascar, BP 1274, Ambohitrakely, 101 Antananarivo, Madagascar

**Keywords:** Anopheles, Urban malaria, Insecticide resistance, kdr-e, Dakar, Senegal

## Abstract

**Background:**

Urban malaria is now considered a major emerging health problem in Africa and urban insecticide resistance may represent a serious threat to the ambitious programme of further scaling-up coverage with long-lasting insecticide-treated bed nets and indoor residual spray. This study evaluates the levels and mechanisms of insecticide resistance in *Anopheles gambiae* populations in 44 urban areas of Dakar in a longitudinal entomological surveillance study.

**Methods:**

Adult mosquitoes sampled by night-landing catches at 44 sites across Dakar from 2007 to 2010 were genotyped to assess the frequency and distribution of resistance alleles. In addition World Health Organization susceptibility tests to six insecticides were performed on F0 adults issuing from immature stages of *An. gambiae* s.l. sampled in August 2010, 2011 and 2012 in three sites of Dakar: Pikine, Thiaroye and Almadies and repeated in 2012 with three of the insecticides after PBO exposure to test for mechanisms of oxydase resistance. Species, molecular forms and the presence of *kdr* and *ace*-*1* mutations were assessed by polymerase chain reaction.

**Results:**

High frequencies of the *kdr*-*e* allele, ranging from 35 to 100 %, were found in *Anopheles arabiensis* at all 44 sites. The insecticide susceptibility tests indicated sensitivity to bendiocarb in Almadies in 2010 and 2011 and in Yarakh between 2010 and 2012 and sensitivity to fenitrothion in Almadies in 2010. The mortality rate of EE genotype mosquitoes was lower and that of SS mosquitoes was higher than that of SE mosquitoes, while the mortality rate of the SW genotype was slightly higher than that of the SE genotype. Pyperonyl butoxide (PBO) had a significant effect on mortality in Pikine (OR = 1.4, 95 % CI = 1.3–1.5, with mortality of 42–55 % after exposure and 11–17 % without PBO) and Yarakh (OR = 1.6, 95 % CI = 1.4–1.7, with mortality of 68–81 % after exposure and 23–37 % without), but not in Almadies (OR = 1.0, 95 % CI = 0.9–1.1).

**Conclusion:**

A high prevalence of *kdr*-*e* in West Africa was demonstrated, and knock-down resistance mechanisms predominate although some oxidases mechanisms (cytochrome P450 monooxygenases) also occur. In view of the increased use of insecticides and the proposed role of the *kdr* gene in the susceptibility of *Anopheles* to *Plasmodium*, this finding will significantly affect the success of vector control programmes.

**Electronic supplementary material:**

The online version of this article (doi:10.1186/s12936-015-0898-6) contains supplementary material, which is available to authorized users.

## Background

Resistance of malaria vectors to insecticides is a major concern for public health authorities and especially for national malaria control programmes in Africa, where the prevention of this devastating disease relies heavily on the use of pesticides to control the vector mosquito populations [1–3]. Currently, pyrethroids are the only class of insecticide approved for treating bed nets, and they are used preferentially to treat long-lasting insecticide-treated bed nets because of their effectiveness, with a strong excito-repellent effect on mosquitoes, and lower mammalian toxicity than organochlorine, carbamate and organophosphate compounds [4].

Various mechanisms enable *Anopheles* to resist the action of insecticides, including metabolic resistance, target-site resistance, reduced penetration and behavioural resistance. These mechanisms may allow mosquitoes to resist more than one insecticide (cross-resistance), and *Anopheles* may express more than one resistance mechanism (multiple resistances). Of all types of resistance, perhaps the most significant in *Anopheles gambiae* populations is knockdown resistance (*kdr*) [5]. Two point mutations at amino acid position 1014 of the voltage-gated sodium channel gene result in either a leucine–phenylalanine (L1014F, *kdr*-*w*) [6] or a leucine–serine (L1014S, *kdr*-*e*) mutation [7] in *An. gambiae* populations mainly in West and East Africa, respectively. A number of studies, with limited geographical sampling, have shown the distribution of *kdr* mutations in *An. gambiae*, with screening for the L1014F allele in West Africa [8–10] and the L1014S mutation in East Africa [7].

Increases in the prevalence of these mutations have been reported, which may be due to intensive use of DDT and pyrethroids for crop protection, particularly cotton, and for public health purposes. Use of pyrethroids in agriculture and for treating nets has been recognized as a factor in the selection of resistant mosquitoes in sub-Saharan Africa [11, 12]. The presence of the two resistance alleles has been studied in Nigeria [13], Benin [14], Cameroon [15], Equatorial Guinea [16], Gabon [17], Uganda [18], Kenya and the United Republic of Tanzania [19], Malawi and Mozambique [20], but so far not in Senegal, the far west of the continent [21].

Malaria is a major public health concern in Senegal. It occurs throughout the year, with a peak during the rainy season. Since 2000, the national malaria control programme has elaborated 5-year action plans, which were successfully implemented with partners [22]. The aim of the latest plan (2006–2010) was to reduce mortality and morbidity from malaria by 50 %. Between the beginning of 2006 and the end of 2009, proportional morbidity due to malaria fell from 33.6 to 3.1 % and proportional mortality from 18.2 to 4.4 % [2, 22]. These results were due to wide-scale treatment of malaria with artemisinin-based combination therapy, generalized use of free rapid diagnostic tests to confirm malaria, massive distribution of long-lasting insecticide-treated bed nets for the most vulnerable people and indoor residual spraying with pyrethroids [23, 24].

Urbanization used to be thought to reduce malaria transmission [25], but this view is changing, and urban malaria is now considered a major emerging health problem in Africa [26]. The rapid, unplanned growth of towns and cities generally results in inferior housing, poor sanitation and increased pollution, all of which could affect the distribution and abundance of mosquito vectors [27, 28]. Invasion of these ‘urban islands’ by malaria vectors, mainly *Anopheles arabiensis*, can be attributed to local adaptation and use of atypical breeding sites, such as polluted water pools or ditches [29, 30]. Urban insecticide resistance might therefore represent a serious threat to the ambitious programme of further scaling-up coverage with long-lasting insecticide-treated bed nets.

No study has been conducted to assess whether urbanization is a threat to malaria control in Senegal. To test this hypothesis, a longitudinal entomological surveillance study was initiated, with monitoring of the level and mechanisms of insecticide resistance in *An. arabiensis* populations at three urban sites near Dakar.

## Methods

### Study area

This study is part of the Urban Malaria Project, which has been described in detail elsewhere [21]. Briefly, Dakar (14°40′20″ North, 17°25′22″ West), the capital city of Senegal, is located on the Cap-Vert peninsula at the western-most point of Africa. The population was estimated at 1,030,594 in 2005, representing about 20 % of the country’s population, with a population density of 12,233 per km^2^. The altitude does not exceed 104 m. The study reported here was conducted in 44 different areas of downtown Dakar and in Pikine, Thiaroye and Guediawaye, three of Dakar’s satellite cities. The urbanization, climate and characteristics of these areas have been described in detail [21, 30, 31].

### Mosquito collection

Adult mosquitoes were collected in the 45 areas of Dakar city by human landing catches once every 2 weeks in September–October 2007, during the extended wet season (July–December) in 2008 and 2009 and once every month during the dry season (January–June) in 2009 and 2010 by indoor and outdoor collections. These mosquitoes were identified and genotyped to assess the frequency and distribution of resistant alleles across the study area.

Immature stages of *An. gambiae* s.l. were collected from 10 breeding sites in three of 44 areas: Almadies, Yarakh and Pikine in August 2010, 2011 and 2012, during the rainy season. Larvae from each area were pooled, fed Tetramin^®^ baby fish food and allowed to emerge locally. These mosquitoes were used for susceptibility tests, and a subset was further identified and genotyped to assess the mechanisms of resistance.

### Susceptibility testing

Unfed 2 to 3-day-old female *An. gambiae* s.l. mosquitoes grown from larvae collected in 2010, 2011 and 2012 were used for insecticide susceptibility tests. Bioassays were carried out with World Health Organization (WHO) test kits for adult mosquitoes [32] on six insecticides of technical-grade quality: one carbamate (0.1 % bendiocarb), one organophosphate (1 % fenitrothion), three pyrethroids (0.05 % λ-cyhalothrin, 0.05 % deltamethrin, 0.75 % permethrin) and one organochlorine (4 % DDT). Papers impregnated with these insecticides were obtained from the WHO reference centre (Vector Control Research Unit, University Sains Malaysia, Penang, Malaysia).

Tests were performed with four batches of 25 mosquitoes exposed to each insecticide for 1 h at 25–27 °C and 80 % relative humidity, and the number of mosquitoes knocked down was recorded after 10, 15, 20, 30, 40, 50 and 60 min. After exposure, the mosquitoes were kept in observation tubes and supplied with a 10 % sugar solution. Mortality was recorded after 24 h. A control strain (*An. gambiae* M form, from Yaoundé, named ‘Boudin’ [24]), which is known to be 100 % susceptible to DDT, was used as a positive control, and batches exposed to untreated paper were used as negative controls. As the mortality rate in negative controls was always <5 %, no adjustment was performed for treated batches. Resistant and susceptible mosquitoes were preserved separately in Eppendorf tubes filled with desiccated silica gel.

### Synergism bioassays

Unfed 2 to 3-day-old female *An. gambiae* s.l. grown from larvae collected in 2012 were used for synergism bioassays. The mosquitoes were exposed to 4 % pyperonyl butoxide (PBO) for 1 h to suppress oxidase resistance mechanisms (cytochrome P450 monooxygenases), leaving only other mechanisms of resistance to be measured. Two batches of 25 mosquitoes were then immediately exposed to 0.05 % deltamethrin, 0.75 % permethrin or 4 % DDT for another 1 h; controls were exposed to PBO only. Knockdown (KDT) was recorded every 10 min during the 1-h exposure. The mosquitoes were then transferred into holding tubes and supplied with a 10 % sugar solution. Final mortality was recorded after 24 h. Resistant and susceptible mosquitoes were preserved separately in Eppendorf tubes filled with desiccated silica gel.

### DNA extraction, molecular identification and detection of mutations

*Kdr*-*w* (L1014F) and *kdr*-*e* (L1014S) mutations and insensitive G119S (*ace*-*1*) point mutations were detected by polymerase chain reaction, as described previously [33, 34], in adult mosquitoes sampled by human landing catch during the longitudinal study and in a subset of the subsample of mosquitoes used for susceptibility testing. This subset was made as fellow: for each insecticide, site and year, 25 mosquitoes were genotyped, of which approximately half were alive and half dead after their exposure within the susceptibility testing. A restriction fragment-length polymorphism assay was also performed to identify members of the *An. gambiae* complex and M and S molecular forms simultaneously [35].

### Data analysis

Four datasets were analysed: (1) the identification and genotypes of the collected adult mosquitoes; (2) the results of susceptibility testing; (2b) the results for the subsample of these mosquitoes that were also genotyped; and (3) the results of the synergism bioassay.

Dataset 1 was used to determine the spatial distribution of the *kdr* mutation.

Dataset 2 to assess levels of resistance and their variations and dataset 2b and 3 their mechanisms.

In order to assess the level of resistance at each site and for each year according to WHO standards, mortality rates was calculated after 24 h and their confidence intervals (CIs). If the mortality rate was <80 %, the mosquitoes were considered resistant; if the rate was 80–98 %, the mosquitoes were considered increasingly tolerant; and if the rate was >98 %, the mosquitoes were considered to be sensitive. In addition, a probit log-time model of which only significant terms had been retained was adjusted on the first hour mortalities (kinetics) to determine 50 and 95 % knockdown times (KDT_50_ and KDT_95_) for each site and each year as well.

In order to assess whether the levels of resistance varied significantly over time and space, a generalized linear model with binomial error structure (logistic regression) was used, with year, site and insecticide as the explanatory variables and 24 h mortality as the response variable. The design was balanced, with 100 mosquitoes per year, site and insecticide (*N* = 5399).

Mechanisms of resistance were assessed from dataset 2b, from the relations between genotypes at the *kdr* and *ace1* loci and mortality, and from dataset 3, which indicated the extent of oxidase resistance.

The sampling method for dataset 2b corresponded to a case–control design, and it was therefore analysed accordingly. Logistic regression was performed with mortality as a response variable, genotype as an explanatory variable and year, site, insecticide and insecticide–site interaction as control variables, as they were shown to affect mortality significantly in analyses of variation in levels of resistance over time and space. A rare event correction [36] was performed (with weights calculated using dataset 2) to allow for interpretable estimates of mortality for the different genotypes despite the case control sampling design. The logistf R library was used to compute the: confidence intervals of these mortalities from the profile-penalized log likelihood. The SE genotype was selected as the baseline for pairwise comparisons because it shares one allele with all the other genotypes (SS, SW and EE). The significance of variables was tested with the drop1 R function.

A logistic model was used on a combined subset of dataset 2 (bioassays) and dataset 3 (synergism assays) after DDT, permethrin and deltamethrin treatment in 2012. The response variable was mortality; the explanatory variables were insecticide, site and exposure to PBO before the insecticide (Yes/No). Type II sum of squares was used to test significance.

## Results

### Spatial distribution of *kdr* mutation

A total of 44,967 mosquitoes were captured between 2007 and 2010 across the 44 sites of Dakar and its three satellite cities. 3753 *An.gambiae* s.l. were identified at species level. *Anopheles arabiensis* represented 93.15 % (n = 3496) of the anophelines and *Anopheles melas* represented 6.84 % (n = 257).

**Fig. 1:**
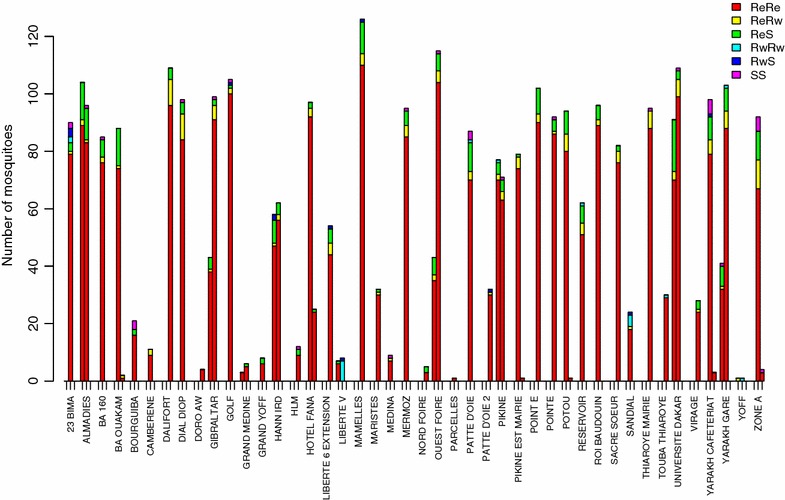
Distribution of *kdr* east and west alleles in *Anopheles arabiensis* populations collected in Dakar (44 sites) between 2007 and 2010: *ReRe* homozygote *kdr* east resistant, *RwRw* homozygote *kdr* west resistant, *SS* homozygote susceptible

The *kdr*-*e* allele was observed in 35–100 % of these mosquitoes at all sites, showing no clear overall spatial pattern (Fig. 1). Of the 44 sites, 41 had a frequency of at least 75 % of the EE genotype; the SE genotype reached a level of about 15 %, particularly in west, in the region of the airport (Additional file 1). In 2007, there is a higher prevalence of ES genotype in the west compared to 2008 and 2009, when the prevalence of EE genotypes was equally high throughout the region (Additional file 1).

### Insecticide resistance

**Fig. 2:**
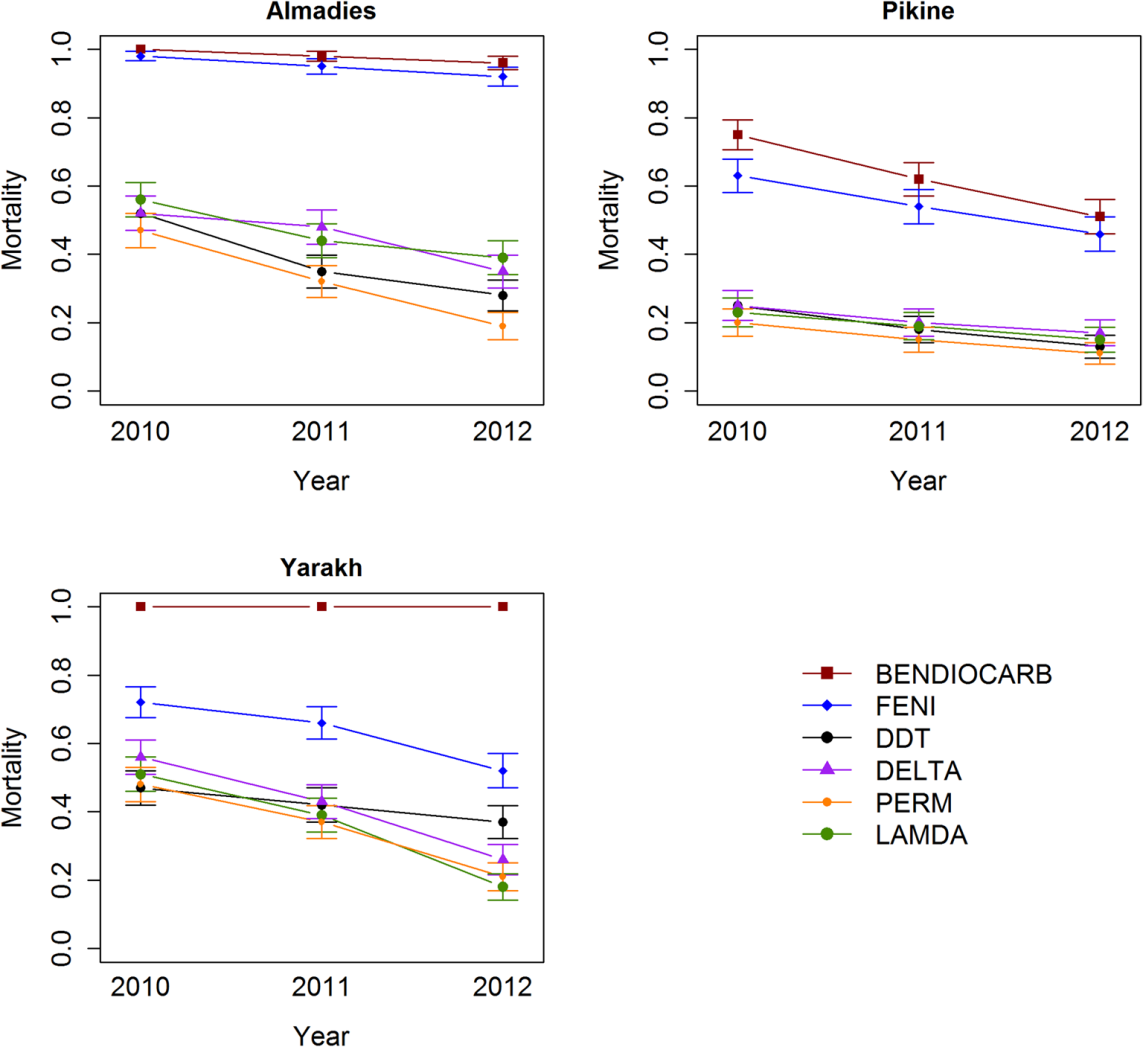
Insecticide-induced mortality in Almadies, Pikine and Yarakh in 2010, 2011 and 2012 per insecticide. Mortality rates expressed as percentages. *Error bars* represent 95 % CI

Sensitivity to bendiocarb was observed in Almadies in 2010 and 2011 and in Yarakh between 2010 and 2012, and to fenitrothion in Almadies in 2010. Increased tolerance was observed to bendiocarb in Almadies in 2013 and in Pikine in 2010, and to fenitrothion in Almadies in 2011 and 2012 and in Yarakh in 2010. Resistance was observed otherwise (i.e. to all other insecticides, year, site) (Fig. 2; Additional file 2). The values of KDT_50_ and KDT_95_ are given for each site and each year in Table 1. These could not be determined for most insecticides at most sites, as mortalities 50 and 95 % were not reached. Insecticide, year and site all had a significant effect on mortality (*p* < 2.2e−16), as did the site–insecticide interaction. The year–insecticide and site–year interactions were not significant (Table 2; Fig. 2; Additional file 2).

**Table 1:** Bioassay susceptibility tests on *Anopheles arabiensis* populations from Almadies, Pikine and Yarakh: evolution of the KD_50_ and KD_95_ by year and by insecticides

Site	Year	Bendiocarb		DDT		Deltamethrin		Fenitrothion		λ-Cyhalothrin		Permethrin	
		KDT_50_	KDT_95_	KDT_50_	KDT_95_	KDT_50_	KDT_95_	KDT_50_	KDT_95_	KDT_50_	KDT_95_	KDT_50_	KDT_95_
Almadies	2010	14	50	–	–	5	–	47	–	37	–	–	–
	2011	14	60	–	–	–	–	46	–	–	–	–	–
	2012	16	–	–	–	–	–	53	–	–	–	–	–
Pikine	2010	–	–	–	–	–	–	–	–	–	–	–	–
	2011	–	–	–	–	–	–	–	–	–	–	–	–
	2012	–	–	–	–	–	–	–	–	–	–	–	–
Yarakh	2010	15	35	–	–	57	–	–	–	–	–	–	–
	2011	16	38		–	–	–		–	–	–	–	–
	2012	21	55	–	–	–	–	–	–	–	–	–	–

–: As no mosquitoes were knocked down, we were unable to measure KDT_50_ and KDT_95_

**Table 2:** Bioassay susceptibility tests in *Anopheles arabiensis* populations from Almadies, Pikine and Yarakh: significance and effect of site, year and insecticide on insecticide-induced mortality

	Df	Deviance	Residual Df	Residual Dev	Pr (>χ^2^)
Null			5398	7476.2	
Site	2	305.20	5396	7171.0	<2e−16
Year	1	102.96	5395	7068.0	<2e−16
Insecticide	5	1277.32	5390	5790.7	<2e−16
Site–insecticide	10	199.02	5378	5590.1	<2e−16
Site–year	2	1.59	5388	5789.1	0.4507
Year–insecticide	5	4.61	5373	5585.5	0.4659
Site–year–insecticide	10	10.48	5363	5575.0	0.3994

### Mechanisms of resistance

All the mosquitoes genotyped and identified are *An*. *arabiensis*. None of them had the *ace*-*1* resistant allele, but *kdr* resistant alleles were found. In the analysis of the relation between insecticide-induced mortality and genotype at the *kdr* locus (dataset 2b), all the explanatory variables, including genotype, were significant (χ^2^ = 68.86, df = 3, p = 7.44e−15). As expected, the mortality rate of EE genotype mosquitoes was lower than that of SE mosquitoes (OR = 0.225, 95 % CI = 0.213–0.239), while the mortality rate of SS mosquitoes was higher (OR = 1418, 95 % CI = 89–22,708). Note the latter OR is highly inaccurate because the mortality rate was close to 1. The mortality rate of the SW genotype was slightly higher than that of the SE genotype (OR = 3.192, 95 % CI = 2.631–3.872). A trend to decreasing mortality was seen between 2010 and 2012 in each locality and for each *kdr* genotype (Additional file 3).

The model used to analyse dataset 3 showed an effect of insecticide and site and their interaction, consistent with our previous results. Moreover, PBO and the interaction between site and PBO exposure had significant effects. Interestingly, the interaction between exposure to PBO and insecticide was not significant (Table 3). As the effect of PBO varied by site, a subset logistic regression was conducted to assess the effect of PBO for each site separately (Table 4). PBO exposure significantly increased mortality among mosquitoes from Pikine (OR = 1.4, 95 % CI = 1.3–1.5) and Yarakh (OR = 1.6, 95 % CI = 1.4–1.7) but not in Almadies (OR = 1.0, 95 % CI = 0.9–1.1).

**Table 3:** Effect of PBO exposure on the susceptibility of *Anopheles arabiensis* populations from Almadies, Pikine and Yarakh: interaction between site, PBO and insecticide

	LR χ^2^	Df	Pr (>χ^2^)	Significance
Insecticide	12.344	2	0.002087	**
Site	40.166	2	1.897e−09	***
PBO	102.936	1	<2e−16	***
Insecticide–site	11.603	4	0.020563	*
Insecticide–PBO	0.665	2	0.717253	
Site–PBO	43.937	2	2.879e−10	***
Insecticide–site–PBO	1.940	4	0.746827	

*** p value < 0.001; ** p value (0.001–0.01); * p value (0.01–0.05)

**Table 4:** Effect of PBO exposure on susceptibility of *Anopheles arabiensis* populations from Almadies, Pikine and Yarakh

	LR χ^2^	Df	Pr (>χ^2^)	Significance
Almadies				
Insecticide	11.5119	2	0.003164	**
PBO exposure	0.0901	1	0.764034	
Pikine				
Insecticide	2.957	2	0.2279	
PBO exposure	75.473	1	<2e−16	***
Yarakh				
Insecticide	9.44	2	0.008915	**
PBO exposure	100.66	1	<2.2e−16	***

*** p value < 0.001; ** p value (0.001–0.01); * p value (0.01–0.05)

**Fig. 3:**
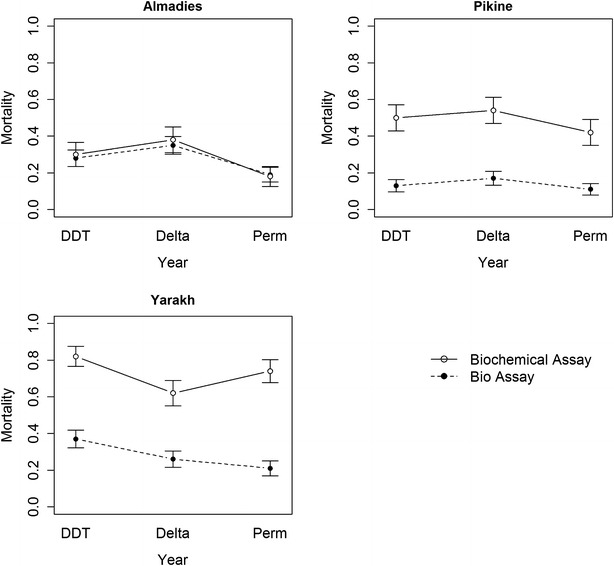
Insecticide-induced mortality of mosquitoes in Almadies, Pikine and Yarakh measured by classical bioassay (*dotted line*) and biochemical assay (*plain line*). *Error bars* represent 95 % CI

For Pikine, mosquito mortality ranged from 42 and 55 %, depending on the insecticide, after PBO exposure but from 11 to 17 % without PBO exposure. For Yarakh, mosquito mortality ranged from 68 to 81 % after PBO exposure but from 23 to 37 % without exposure. For Almadies, mosquito mortality was 18–35 %, independently of PBO exposure (Fig. 3; Additional files 2, 4).

## Discussion

Mosquitoes were resistant to most of the insecticides at most sites. Resistance to bendiocarb appeared to have progressed less than that to other insecticides, as no resistance to this insecticide was found in 2010. Mosquitoes in Yarakh remained sensitive until 2012, but those in Pikine were significantly resistant and those in Almadies showed increased tolerance to this insecticide up to 2011. The KDT values in response to fenitrothion and bendiocarb were similar in 2010 and 2011 but increased markedly between 2011 and 2012, indicating accelerated change. The impossibility to determine KDT_50_ and KDT_95_ for most molecules indicates high levels of resistances, in particular knockdown resistance.

The level of resistance to the different insecticides varied geographically, as indicated by the significant effect of the insecticide–site interaction on insecticide-induced mortality. The significance of the year variable, with no interaction with site or insecticide, indicates a trend to decreasing mortality (increasing resistance) over time. The genotype at the *kdr* locus affects the mosquito’s insecticide-induced mortality, indicating the involvement of the *kdr* locus in the resistance of mosquitoes in Dakar. The trend to decreasing mortality within *kdr* genotypes between 2010 and 2012, however, suggests that a further resistance mechanism might have evolved between those dates.

This study shows for the first time the presence of a high rate of *kdr*-*e* mutations in Senegal and an increase in frequency over time. Previously, this mutation was observed only in East Africa [7], whereas it now appears to be invading West Africa [14], with the direct consequence of the disappearance of the barrier between *kdr*-*e* and *kdr*-*w*, allowing significant gene flow among the different populations of anopheles and among bio-geographical regions.

The insecticide-induced mortality of SW genotypes was slightly higher than that of SE genotypes, which suggests that natural selection favours SE genotypes over SW genotypes and could explain the rapid increase in the frequency of E alleles. This study demonstrated a significant effect of *kdr* locus on mortality, SS genotypes being more sensitive than SE genotypes, SW genotypes being intermediary between SS and SE genotypes and EE genotypes being less sensitive than the other genotypes.

Djegbe et al. [14] described the presence of the *kdr*-*e* allele in anopheles populations in West Africa for the first time in Benin, in 2011. Those authors found a low level of circulation of this allele, which was, however, sufficient as an indicator of diffusion. A part from *kdr*-type resistance, our study also showed physiological resistance of *Anopheles* to pyrethroids and DDT in all three neighbourhoods of Dakar studied, which increased over the years. In some areas, resistance to organophosphates and carbamates was exacerbated; however, even if the presence of the *kdr* allele results, to a large extent, from the use of pesticides in the Niayes area [37], this cannot explain its high prevalence throughout the region, especially in coastal areas and in residential areas such as Almadies. Massive unplanned aerial spraying with insecticide should also be taken into consideration. As the frequency of *kdr*-*e* was much higher in Dakar than in Benin, diffusion of *kdr*-*e* to the interior of the continent in this part of West Africa could gather momentum. Additional studies are needed to test this hypothesis as well as the mechanisms of diffusion.

Strong resistance to insecticides is important for many reasons. Recent studies have shown that *kdr*-type resistance could seriously compromise the effectiveness of insecticide-treated nets [23, 38], and the presence of *kdr* mutations in *Anopheles* may significantly increase their susceptibility to *Plasmodium* infection [39]. The Senegalese national malaria control programme has embarked on a comprehensive initiative to provide nets for every bed, except in the Dakar region. Our results put into question this action. The presence of the *kdr*-*e* allele, associated with high physiological and metabolic resistance to pyrethroids, could have a negative impact on vector control.

The finding that the mortality of mosquitoes in Pikine and Yarakh differed significantly with exposure to PBO before another insecticide indicates that these mosquitoes have an oxidase resistance mechanism. In contrast, the mortality rate of mosquitoes in Almadies was the same independently of their exposure to PBO, suggesting the absence of an oxidase resistance mechanism (cytochrome P450 monooxygenases). A significant oxidase resistance mechanism was seen in Yarakh and Pikine. These observations could explain why a given *kdr* genotype results in different mortality depending on site, and selection of a mechanism could explain why mortality decreases for a given *kdr* genotype over time (Fig. 3).

Resistance to fenitrothion and tolerance to bendiocarb have gradually begun to be seen in the Dakar region. Although a high frequency of *ace1* was not found, further studies should be conducted to identify any other biochemical intermediate, such as esterases or glutathione-S-transferase. The various types of resistance observed in *Anopheles* populations [5, 40] could have disastrous consequences on the long road towards malaria eradication.

## Conclusion

Widespread use of insecticides in indoor residual spraying and long-lasting insecticide-treated bed nets has a mixed role in the spread of the *kdr* gene. If behavioural resistance is added, there is no doubt that the combination of these forms of resistance will slow or significantly alter vector control. There is room for hope, however, because, despite widespread resistance, the prevalence of malaria in Senegal has never been so low. The efforts that led to this result must be both sustained and maintained.

## Additional files

**Additional file 1.** Evolution of kdr frequency in *Anopheles arabiens* populations collected in 44 sites in Dakar between 2007 to 2009.
**Additional file 2.** Insecticide-induced mortality of mosquitoes in Pikine, Yarakh and Almadies, with 95% confidence intervals. “Sens.” indicates sensitivity (mortality >98%), “I.T.” indicates increased tolerance (mortality 80–98%), “Res.” indicates resistance (mortality <80%).
**Additional file 3.** Mortality predicted in the model for each genotype at each site, for each insecticide, each year. Several genotypes were not found at some sites; in particular, the SW genotype was found only in Yarakh.
**Additional file 4.** Insecticide-induced mortality of mosquitoes in Pikine, Yarakh and Almadies with and without prior exposure to PBO.
