# Supplementary material for: Emerging knock-down resistance in Anopheles arabiensis populations of Dakar, Senegal: first evidence of a high prevalence of kdr-e mutation in West African urban area
Source: Malar J. 2015 Sep 22;14:364. doi: 10.1186/s12936-015-0898-6 (PMC4579585; doi:10.1186/s12936-015-0898-6)
Supplement: Supplementary file 1 — Additional file 1. Evolution of kdr frequency in Anopheles arabiens populations collected in 44 sites in Dakar between 2007 to 2009. [file 12936_2015_898_MOESM1_ESM.pdf]

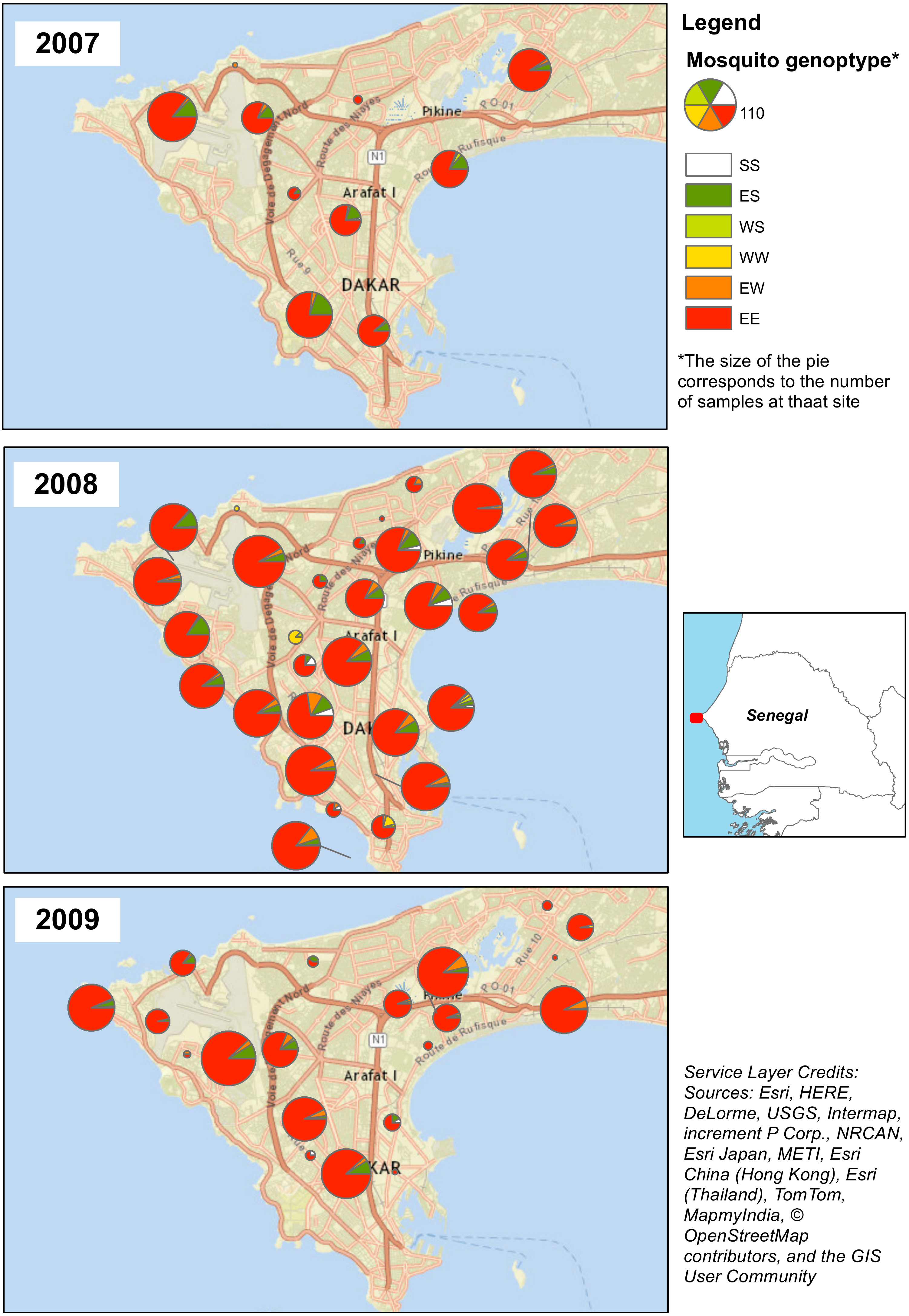
2007
Pikine
Arafat I
DAKAR
N1
Legend
Mosquito genoptype*
110
SS
ES
WS
WW
EW
EE
*The size of the pie corresponds to the number of samples at thaat site
2008
Pikine
Arafat I
Senegal
2009
Arafat I
N1
Service Layer Credits: Sources: Esri, HERE, DeLorme, USGS, Intermap, increment P Corp., NRCAN, Esri Japan, METI, Esri China (Hong Kong), Esri (Thailand), TomTom, MapmyIndia, © OpenStreetMap contributors, and the GIS User Community
