# Supplementary material for: Emerging knock-down resistance in Anopheles arabiensis populations of Dakar, Senegal: first evidence of a high prevalence of kdr-e mutation in West African urban area
Source: Malar J. 2015 Sep 22;14:364. doi: 10.1186/s12936-015-0898-6 (PMC4579585; doi:10.1186/s12936-015-0898-6)
Supplement: Supplementary file 2 — Additional file 2. Insecticide-induced mortality of mosquitoes in Pikine, Yarakh and Almadies, with 95% confidence intervals. “Sens.” indicates sensitivity (mortality >98%), “I.T.” indicates increased tolerance (mortality 80–98%), “Res.” indicates resistance (mortality <80%). [file 12936_2015_898_MOESM2_ESM.docx]

| **Place** | **Year** | **Molecule** | **Mortality** | **Confidence interval LL** | **Confidence Interval UL** | **Diagnosis** |
| --- | --- | --- | --- | --- | --- | --- |
| Almadies | 2010 | BENDIO | 100 | 100 | 100 | Sens. |
| Almadies | 2011 | BENDIO | 98 | 95 | 100 | Sens. |
| Almadies | 2012 | BENDIO | 96 | 92 | 100 | I.T. |
| Almadies | 2010 | DDT | 52 | 42 | 62 | Res. |
| Almadies | 2011 | DDT | 35 | 26 | 44 | Res. |
| Almadies | 2012 | DDT | 28 | 19 | 37 | Res. |
| Almadies | 2010 | DELTA | 52 | 42 | 62 | Res. |
| Almadies | 2011 | DELTA | 48 | 38 | 58 | Res. |
| Almadies | 2012 | DELTA | 35 | 26 | 44 | Res. |
| Almadies | 2010 | FENI | 98 | 95 | 100 | Sens. |
| Almadies | 2011 | FENI | 95 | 91 | 99 | I.T. |
| Almadies | 2012 | FENI | 92 | 87 | 97 | I.T. |
| Almadies | 2010 | LAMDA | 56 | 46 | 66 | Res. |
| Almadies | 2011 | LAMDA | 44 | 34 | 54 | Res. |
| Almadies | 2012 | LAMDA | 39 | 29 | 49 | Res. |
| Almadies | 2010 | PERM | 47 | 37 | 57 | Res. |
| Almadies | 2011 | PERM | 32 | 23 | 41 | Res. |
| Almadies | 2012 | PERM | 19 | 11 | 27 | Res. |
|  |  |  |  |  |  |  |
| Pikine | 2010 | BENDIO | 75 | 66 | 84 | I.T. |
| Pikine | 2011 | BENDIO | 62 | 52 | 72 | Res. |
| Pikine | 2012 | BENDIO | 51 | 41 | 61 | Res. |
| Pikine | 2010 | DDT | 25 | 16 | 34 | Res. |
| Pikine | 2011 | DDT | 18 | 10 | 26 | Res. |
| Pikine | 2012 | DDT | 13 | 6 | 20 | Res. |
| Pikine | 2010 | DELTA | 25 | 16 | 34 | Res. |
| Pikine | 2011 | DELTA | 20 | 12 | 28 | Res. |
| Pikine | 2012 | DELTA | 17 | 10 | 24 | Res. |
| Pikine | 2010 | FENI | 63 | 53 | 73 | Res. |
| Pikine | 2011 | FENI | 54 | 44 | 64 | Res. |
| Pikine | 2012 | FENI | 46 | 36 | 56 | Res. |
| Pikine | 2010 | LAMDA | 23 | 15 | 31 | Res. |
| Pikine | 2011 | LAMDA | 19 | 11 | 27 | Res. |
| Pikine | 2012 | LAMDA | 15 | 8 | 22 | Res. |
| Pikine | 2010 | PERM | 20 | 12 | 28 | Res. |
| Pikine | 2011 | PERM | 15 | 8 | 22 | Res. |
| Pikine | 2012 | PERM | 11 | 5 | 17 | Res. |
|  |  |  |  |  |  |  |
| Yarakh | 2010 | BENDIO | 100 | 100 | 100 | Sens. |
| Yarakh | 2011 | BENDIO | 100 | 100 | 100 | Sens. |
| Yarakh | 2012 | BENDIO | 100 | 100 | 100 | Sens. |
| Yarakh | 2010 | DDT | 47 | 37 | 57 | Res. |
| Yarakh | 2011 | DDT | 42 | 32 | 52 | Res. |
| Yarakh | 2012 | DDT | 37 | 27 | 47 | Res. |
| Yarakh | 2010 | DELTA | 56 | 46 | 66 | Res. |
| Yarakh | 2011 | DELTA | 43 | 33 | 53 | Res. |
| Yarakh | 2012 | DELTA | 26 | 17 | 35 | Res. |
| Yarakh | 2010 | FENI | 72 | 63 | 81 | I.T. |
| Yarakh | 2011 | FENI | 66 | 57 | 75 | Res. |
| Yarakh | 2012 | FENI | 52 | 42 | 62 | Res. |
| Yarakh | 2010 | LAMDA | 51 | 41 | 61 | Res. |
| Yarakh | 2011 | LAMDA | 39 | 29 | 49 | Res. |
| Yarakh | 2012 | LAMDA | 18 | 10 | 26 | Res. |
| Yarakh | 2010 | PERM | 48 | 38 | 58 | Res. |
| Yarakh | 2011 | PERM | 37 | 27 | 47 | Res. |
| Yarakh | 2012 | PERM | 21 | 13 | 29 | Res. |

**Additional file 2**: Insecticide-induced mortalities of mosquitoes from Pikine, Yarakh, and Almadies, with the 95% confidence intervals. “Sens.” indicates sensitivity (mortality > 98%), “I.T.” indicates increased tolerance (mortality between 80% and 98%), “Res.” Indicates resistance (mortality below 80%).
