# Supplementary material for: Emerging knock-down resistance in Anopheles arabiensis populations of Dakar, Senegal: first evidence of a high prevalence of kdr-e mutation in West African urban area
Source: Malar J. 2015 Sep 22;14:364. doi: 10.1186/s12936-015-0898-6 (PMC4579585; doi:10.1186/s12936-015-0898-6)
Supplement: Supplementary file 3 — Additional file 3. Mortality predicted in the model for each genotype at each site, for each insecticide, each year. Several genotypes were not found at some sites; in particular, the SW genotype was found only in Yarakh. [file 12936_2015_898_MOESM3_ESM.docx]

| **Annee** | **Lieu** | **Molecule** | **Genotype** | **Predicted Mortalities** |  | **Annee** | **Lieu** | **Molecule** | **Genotype** | **Predicted Mortalities** |
| --- | --- | --- | --- | --- | --- | --- | --- | --- | --- | --- |
| 2010 | Almadies | BENDIO | EE | 98,5 |  | 2010 | Almadies | FENI | EE | 96,4 |
| 2011 | Almadies | BENDIO | EE | 97,9 |  | 2011 | Almadies | FENI | EE | 95,1 |
| 2012 | Almadies | BENDIO | EE | 97,0 |  | 2012 | Almadies | FENI | EE | 93,0 |
| 2010 | Almadies | BENDIO | SE | 99,7 |  | 2011 | Almadies | FENI | SE | 98,9 |
| 2011 | Almadies | BENDIO | SE | 99,5 |  | 2012 | Almadies | FENI | SE | 98,3 |
| 2012 | Almadies | BENDIO | SE | 99,3 |  | 2010 | Almadies | FENI | SS | 100,0 |
| 2011 | Almadies | BENDIO | SS | 100,0 |  | 2010 | Pikine | FENI | EE | 60,8 |
| 2010 | Pikine | BENDIO | EE | 68,6 |  | 2011 | Pikine | FENI | EE | 52,9 |
| 2011 | Pikine | BENDIO | EE | 61,2 |  | 2012 | Pikine | FENI | EE | 43,4 |
| 2012 | Pikine | BENDIO | EE | 51,9 |  | 2010 | Pikine | FENI | SE | 87,3 |
| 2010 | Pikine | BENDIO | SE | 90,7 |  | 2011 | Pikine | FENI | SE | 83,3 |
| 2011 | Pikine | BENDIO | SE | 87,5 |  | 2010 | Pikine | FENI | SS | 100,0 |
| 2010 | Pikine | BENDIO | SS | 100,0 |  | 2010 | Yarakh | FENI | EE | 66,6 |
| 2011 | Pikine | BENDIO | SS | 100,0 |  | 2011 | Yarakh | FENI | EE | 59,1 |
| 2010 | Yarakh | BENDIO | EE | 100,0 |  | 2012 | Yarakh | FENI | EE | 49,6 |
| 2011 | Yarakh | BENDIO | EE | 100,0 |  | 2010 | Yarakh | FENI | SE | 89,9 |
| 2012 | Yarakh | BENDIO | EE | 100,0 |  | 2011 | Yarakh | FENI | SE | 86,5 |
| 2010 | Yarakh | BENDIO | SE | 100,0 |  | 2012 | Yarakh | FENI | SE | 81,4 |
| 2011 | Yarakh | BENDIO | SE | 100,0 |  | 2010 | Yarakh | FENI | SS | 100,0 |
| 2012 | Yarakh | BENDIO | SE | 100,0 |  | 2011 | Yarakh | FENI | SS | 100,0 |
| 2010 | Yarakh | BENDIO | SS | 100,0 |  | 2011 | Yarakh | FENI | SW | 95,3 |
| 2011 | Yarakh | BENDIO | SS | 100,0 |  | 2010 | Almadies | LAMDA | EE | 50,7 |
| 2010 | Yarakh | BENDIO | SW | 100,0 |  | 2011 | Almadies | LAMDA | EE | 42,6 |
| 2011 | Yarakh | BENDIO | SW | 100,0 |  | 2012 | Almadies | LAMDA | EE | 33,7 |
| 2012 | Yarakh | BENDIO | SW | 100,0 |  | 2010 | Almadies | LAMDA | SE | 82,0 |
| 2010 | Almadies | DDT | EE | 43,1 |  | 2011 | Almadies | LAMDA | SE | 76,7 |
| 2011 | Almadies | DDT | EE | 35,4 |  | 2012 | Almadies | LAMDA | SE | 69,2 |
| 2012 | Almadies | DDT | EE | 27,3 |  | 2010 | Almadies | LAMDA | SS | 100,0 |
| 2010 | Almadies | DDT | SE | 77,1 |  | 2010 | Pikine | LAMDA | EE | 22,1 |
| 2011 | Almadies | DDT | SE | 70,9 |  | 2011 | Pikine | LAMDA | EE | 17,0 |
| 2010 | Almadies | DDT | SS | 100,0 |  | 2012 | Pikine | LAMDA | EE | 12,3 |
| 2010 | Pikine | DDT | EE | 21,1 |  | 2010 | Pikine | LAMDA | SE | 55,7 |
| 2011 | Pikine | DDT | EE | 16,2 |  | 2011 | Pikine | LAMDA | SE | 47,6 |
| 2012 | Pikine | DDT | EE | 11,7 |  | 2012 | Pikine | LAMDA | SE | 38,3 |
| 2010 | Pikine | DDT | SE | 54,3 |  | 2011 | Pikine | LAMDA | SS | 99,9 |
| 2011 | Pikine | DDT | SE | 46,2 |  | 2010 | Yarakh | LAMDA | EE | 37,9 |
| 2012 | Pikine | DDT | SE | 37,0 |  | 2011 | Yarakh | LAMDA | EE | 30,6 |
| 2010 | Yarakh | DDT | EE | 46,6 |  | 2012 | Yarakh | LAMDA | EE | 23,2 |
| 2011 | Yarakh | DDT | EE | 38,7 |  | 2010 | Yarakh | LAMDA | SE | 73,1 |
| 2012 | Yarakh | DDT | EE | 30,1 |  | 2011 | Yarakh | LAMDA | SE | 66,2 |
| 2010 | Yarakh | DDT | SE | 79,5 |  | 2010 | Yarakh | LAMDA | SS | 100,0 |
| 2011 | Yarakh | DDT | SE | 73,7 |  | 2010 | Yarakh | LAMDA | SW | 89,6 |
| 2010 | Yarakh | DDT | SS | 100,0 |  | 2011 | Yarakh | LAMDA | SW | 86,2 |
| 2011 | Yarakh | DDT | SS | 100,0 |  | 2012 | Yarakh | LAMDA | SW | 81,0 |
| 2010 | Yarakh | DDT | SW | 92,5 |  | 2010 | Almadies | PERM | EE | 35,8 |
| 2011 | Yarakh | DDT | SW | 89,9 |  | 2011 | Almadies | PERM | EE | 28,7 |
| 2010 | Almadies | DELTA | EE | 51,1 |  | 2012 | Almadies | PERM | EE | 21,6 |
| 2011 | Almadies | DELTA | EE | 43,1 |  | 2010 | Almadies | PERM | SE | 71,2 |
| 2012 | Almadies | DELTA | EE | 34,1 |  | 2011 | Almadies | PERM | SE | 64,1 |
| 2010 | Almadies | DELTA | SE | 82,3 |  | 2012 | Almadies | PERM | SE | 54,9 |
| 2011 | Almadies | DELTA | SE | 77,1 |  | 2011 | Almadies | PERM | SS | 100,0 |
| 2012 | Almadies | DELTA | SE | 69,6 |  | 2010 | Pikine | PERM | EE | 17,5 |
| 2010 | Almadies | DELTA | SS | 100,0 |  | 2011 | Pikine | PERM | EE | 13,3 |
| 2010 | Pikine | DELTA | EE | 24,7 |  | 2012 | Pikine | PERM | EE | 9,5 |
| 2011 | Pikine | DELTA | EE | 19,2 |  | 2010 | Pikine | PERM | SE | 48,5 |
| 2012 | Pikine | DELTA | EE | 14,0 |  | 2011 | Pikine | PERM | SE | 40,5 |
| 2010 | Pikine | DELTA | SE | 59,3 |  | 2012 | Pikine | PERM | SE | 31,7 |
| 2011 | Pikine | DELTA | SE | 51,3 |  | 2010 | Pikine | PERM | SS | 99,9 |
| 2012 | Pikine | DELTA | SE | 41,9 |  | 2011 | Pikine | PERM | SS | 99,9 |
| 2010 | Pikine | DELTA | SS | 100,0 |  | 2010 | Yarakh | PERM | EE | 34,7 |
| 2010 | Yarakh | DELTA | EE | 43,7 |  | 2011 | Yarakh | PERM | EE | 27,7 |
| 2011 | Yarakh | DELTA | EE | 35,9 |  | 2012 | Yarakh | PERM | EE | 20,8 |
| 2012 | Yarakh | DELTA | EE | 27,7 |  | 2010 | Yarakh | PERM | SE | 70,2 |
| 2010 | Yarakh | DELTA | SE | 77,5 |  | 2011 | Yarakh | PERM | SE | 63,0 |
| 2011 | Yarakh | DELTA | SE | 71,3 |  | 2012 | Yarakh | PERM | SE | 53,8 |
| 2012 | Yarakh | DELTA | SE | 62,9 |  | 2010 | Yarakh | PERM | SS | 100,0 |
| 2010 | Yarakh | DELTA | SS | 100,0 |  | 2011 | Yarakh | PERM | SW | 84,5 |
| 2011 | Yarakh | DELTA | SS | 100,0 |  | 2012 | Yarakh | PERM | SW | 78,8 |
| 2010 | Yarakh | DELTA | SW | 91,6 |  |  |  |  |  |  |
| 2011 | Yarakh | DELTA | SW | 88,8 |  |  |  |  |  |  |
| 2012 | Yarakh | DELTA | SW | 84,4 |  |  |  |  |  |  |

**Additional file 3**: Mortalities predicted by the model for each genotype in each location and for each molecule, each year. Note several genotypes never were found in some localities. In particular, SW genotype was only found in Yarakh.
