# Supplementary material for: Emerging knock-down resistance in Anopheles arabiensis populations of Dakar, Senegal: first evidence of a high prevalence of kdr-e mutation in West African urban area
Source: Malar J. 2015 Sep 22;14:364. doi: 10.1186/s12936-015-0898-6 (PMC4579585; doi:10.1186/s12936-015-0898-6)
Supplement: Supplementary file 4 — Additional file 4. Insecticide-induced mortality of mosquitoes in Pikine, Yarakh and Almadies with and without prior exposure to PBO. [file 12936_2015_898_MOESM4_ESM.docx]

|  |  | **PBO Exposure** | | | **No PBO exposure** | | |
| --- | --- | --- | --- | --- | --- | --- | --- |
| **Lieu** | **Molecule** | **PRED** | **CI1** | **CI2** | **PRED** | **CI1** | **CI2** |
| Almadies | Delta | 37 | 27 | 48 | 35 | 28 | 44 |
| Almadies | Perm | 19 | 13 | 28 | 18 | 13 | 26 |
| Almadies | DDT | 30 | 21 | 40 | 28 | 21 | 37 |
| Pikine | Delta | 55 | 43 | 66 | 17 | 11 | 24 |
| Pikine | Perm | 42 | 32 | 54 | 11 | 7 | 17 |
| Pikine | DDT | 49 | 38 | 60 | 14 | 9 | 20 |
| Yarakh | Delta | 68 | 57 | 77 | 23 | 17 | 31 |
| Yarakh | Perm | 69 | 58 | 78 | 24 | 17 | 32 |
| Yarakh | DDT | 81 | 72 | 87 | 37 | 29 | 46 |

**Additional file 4**: Insecticide-induced mortalities of mosquitoes from Pikine, Yarakh, and Almadies with and without prior exposure to PBO.
